# Elongated inflamed uvula mimicking early recurrence on planning CT in post-laryngectomy laryngeal squamous cell carcinoma: a diagnostic pitfall in adjuvant radiotherapy

**DOI:** 10.1093/omcr/omag057

**Published:** 2026-05-24

**Authors:** Rania Bouanane, Ouiam Taibi, Salma El Aouadi, Fatima Zahra Laamrani, Youssef Omor, Rachida Latib, Sanae Amalik, Ihsane Skitioui, Salma Ichou, Amina Majdi, Amine Lachgar, Karima Nouni, Hanan ElKacemi, Tayeb Kebdani, Khalid Hassouni

**Affiliations:** Department of Radiology, National Institute of Oncology, Faculty of medicine and pharmacy of Rabat, Rabat, Morocco; Department of Radiology, National Institute of Oncology, Faculty of medicine and pharmacy of Rabat, Rabat, Morocco; Department of Radiology, National Institute of Oncology, Faculty of medicine and pharmacy of Rabat, Rabat, Morocco; Department of Radiology, National Institute of Oncology, Faculty of medicine and pharmacy of Rabat, Rabat, Morocco; Department of Radiology, National Institute of Oncology, Faculty of medicine and pharmacy of Rabat, Rabat, Morocco; Department of Radiology, National Institute of Oncology, Faculty of medicine and pharmacy of Rabat, Rabat, Morocco; Department of Radiology, National Institute of Oncology, Faculty of medicine and pharmacy of Rabat, Rabat, Morocco; Department of Radiotherapy, National Institute of Oncology, Faculty of medicine and pharmacy of Rabat, Rabat, Morocco; Department of Radiotherapy, National Institute of Oncology, Faculty of medicine and pharmacy of Rabat, Rabat, Morocco; Department of Radiotherapy, National Institute of Oncology, Faculty of medicine and pharmacy of Rabat, Rabat, Morocco; Department of Radiotherapy, National Institute of Oncology, Faculty of medicine and pharmacy of Rabat, Rabat, Morocco; Department of Radiotherapy, National Institute of Oncology, Faculty of medicine and pharmacy of Rabat, Rabat, Morocco; Department of Radiotherapy, National Institute of Oncology, Faculty of medicine and pharmacy of Rabat, Rabat, Morocco; Department of Radiotherapy, National Institute of Oncology, Faculty of medicine and pharmacy of Rabat, Rabat, Morocco; Department of Radiotherapy, National Institute of Oncology, Faculty of medicine and pharmacy of Rabat, Rabat, Morocco

**Keywords:** elongated uvula, laryngeal squamous cell carcinoma (SCC), post-operative imaging, head and neck imaging pitfalls, false-positive recurrence, post-laryngectomy surveillance

## Abstract

Background: Laryngeal squamous cell carcinoma (SCC) requires rigorous post-operative imaging surveillance to detect early recurrence, yet benign post-surgical changes may mimic malignancy and create diagnostic uncertainty. Case Presentation: We report a case of a 65-year-old male with well-differentiated laryngeal SCC who developed a suspicious lesion in the oropharynx on planning CT for adjuvant radiotherapy, 2 months after total laryngectomy, raising concern for early local recurrence. Clinical Discussion: Further assessment using multiplanar MRI demonstrated a well-defined elongated uvula with homogeneous enhancement, slightly hyperintense on T2 and without diffusion restriction, consistent with postoperative inflammatory change. Comparison with pre-treatment imaging confirmed its benign nature, preventing unnecessary biopsy or PET-CT. Conclusion: This case highlights the importance of distinguishing benign anatomical variants from malignant recurrence during post-operative surveillance imaging in head and neck oncology.

## Introduction

Laryngeal cancer represents a significant global health burden, with approximately 189 000 new cases and more than 103 000 deaths worldwide, and a five-year prevalence of nearly 584 000 patients [1].

Smoking and alcohol consumption remain significant risk factors. Human papillomavirus (HPV), particularly HPV-16, has also been implicated in a subset of head and neck squamous cell carcinomas, although its role in laryngeal cancer remains less clearly defined than in oropharyngeal tumors. Standard treatment for advanced-stage laryngeal carcinoma typically involves surgical resection, often followed by radiotherapy or concurrent chemoradiotherapy, in line with current National Comprehensive Cancer Network (NCCN) 2025 guidelines [2]. Post-operative imaging plays a crucial role in the surveillance of recurrence, but it can also present diagnostic challenges, particularly in distinguishing benign post-surgical findings from true malignancy. This case presents an example of such a challenge, where an elongated uvula mimicked tumor recurrence in a patient following total laryngectomy, emphasising the necessity of thorough imaging analysis and clinical correlation.

## Case report

A 65-year-old male with a 30 pack-year smoking history and occasional alcohol consumption was referred to our institution for postoperative management of laryngeal squamous cell carcinoma (SCC). His medical history included well-controlled hypertension treated with amlodipine. One year prior to presentation, he developed intermittent dysphonia. Flexible fiberoptic laryngoscopy demonstrated subtle irregularity of the true vocal cords without airway compromise, and conservative management was initiated. Six months later, he presented with rapidly progressive dyspnea and noisy breathing. Clinical examination revealed grade III inspiratory stridor and oxygen saturation of 85% on room air, prompting urgent tracheotomy. Direct laryngoscopy with biopsy confirmed well-differentiated SCC involving the true vocal cords and epiglottis.

**Figure 1:**
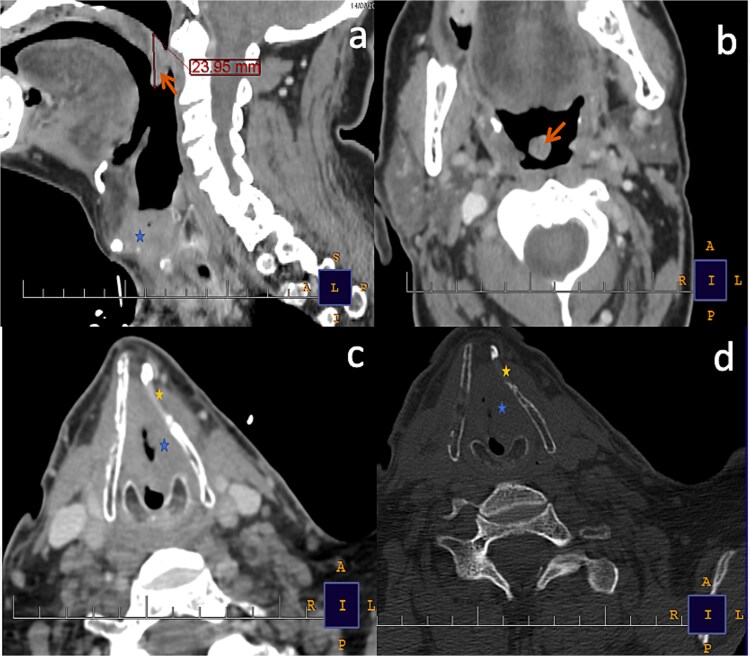
(a) Sagittal and (b) axial contrast-enhanced CT images (soft-tissue window) demonstrate a laryngeal mass centered at the glottic level (blue asterisk), extending to the anterior commissure and causing significant narrowing of the laryngeal lumen. On contrast-enhanced CT, the tumor shows mild heterogeneous enhancement, with attenuation values measuring approximately 65–75 Hounsfield units (HU).The uvula (orange arrow) demonstrates mild, homogeneous contrast enhancement, with attenuation values of approximately 45–50 HU, and measures 23.95 mm in craniocaudal length. (c) Axial contrast-enhanced CT image (soft-tissue window) and (d) axial CT image (bone window) reveal focal lysis of the left thyroid cartilage (yellow asterisk), consistent with tumoral extension from the primary glottic lesion (blue asterisk).

Contrast-enhanced neck CT revealed a 25 × 17 × 26 mm enhancing laryngeal mass partially obstructing the airway with erosion of the arytenoid cartilage and invasion of the thyroid cartilage (Fig. 1). The tumor was staged T3N0M0 according to the AJCC 8th edition classification and measured 26 mm along its longest axis per RECIST 1.1. The patient underwent total laryngectomy with bilateral neck dissection in June 2024. Histopathology confirmed well-differentiated SCC with a close lateral margin (<1 mm), no vascular or perineural invasion, and no nodal metastasis (0/22 nodes), corresponding to stage pT3 pN0. Based on the close margin and cartilage invasion, adjuvant chemoradiotherapy was recommended.

Postoperative radiotherapy planning was performed using VMAT according to consensus postoperative contouring guidelines (Grégoire et al., DAHANCA-EORTC-GORTEC-TROG), employing a simultaneous integrated boost delivering 66/60/54 Gy to primary and elective target volumes over 33 fractions, with concurrent weekly cisplatin (40 mg/m^2^) and standard organ-at-risk dose constraints.

During planning CT, an unexpected newly detected nodular thickening was observed within the oropharynx, raising concern for early tumor recurrence. Prompting further clinical and radiologic assessment.

**Figure 2:**
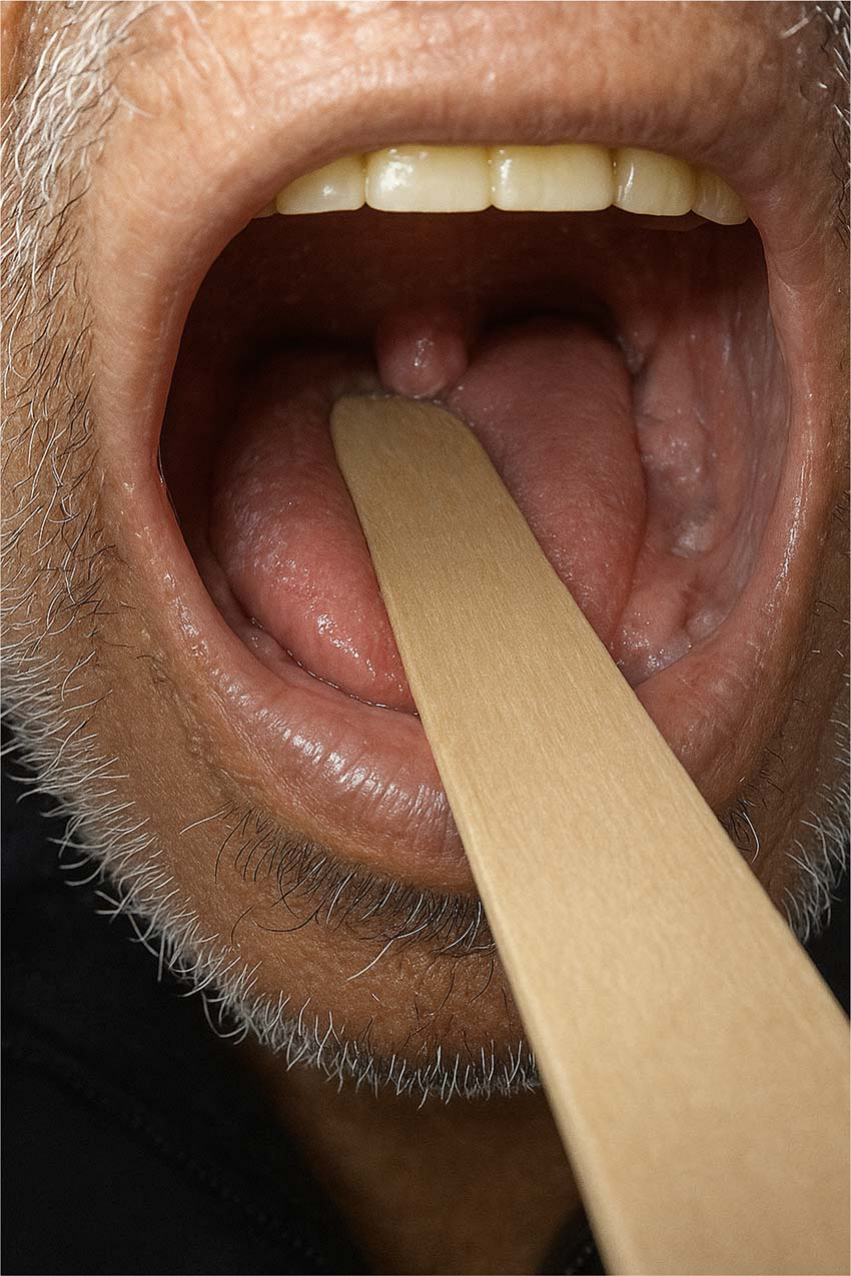
Oropharyngeal inspection using a tongue depressor demonstrates a swollen but non-inflamed uvula, with normal mucosal coloration and no evidence of local infection.

Clinically, examination demonstrated a swollen but non-inflamed uvula, with normal mucosal coloration and no evidence of local infection (Fig. 2). The patient reported only mild swallowing discomfort without fever or odynophagia. Biological markers were normal (CRP and leukocyte count). Flexible endoscopy showed a smooth mucosal surface without ulceration or suspicious focal mass, lowering the suspicion for recurrent disease.

**Figure 3:**
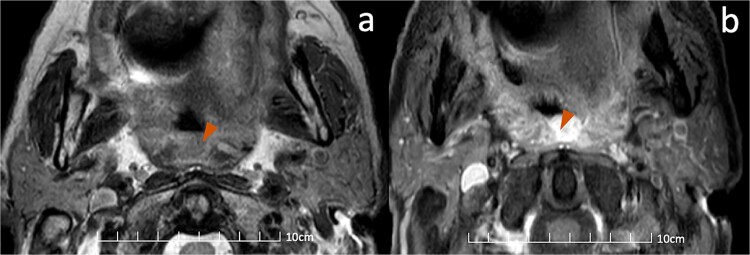
(a) Axial T2: Rounded, isointense mass in the oropharynx, well-delineated with no mass effect (orange arrow). (b) Axial T1 post-contrast: Well-defined oropharyngeal mass with intense, homogeneous enhancement (orange arrow). No adhesion to adjacent structures.

MRI was subsequently performed for clarification. The lesion appeared slightly hyperintense on T2-weighted sequences without diffusion restriction and demonstrated elevated ADC values (1.50 × 10^−3^ mm^2^/s), consistent with a benign inflammatory process. Post-contrast T1-weighted imaging revealed a well-defined, non-adherent structure with homogeneous enhancement corresponding to an elongated uvula (Fig. 3), without discrete soft-tissue mass.

A structured differential diagnosis included recurrent SCC, minor salivary gland tumor, squamous papilloma, post-treatment lymphoid hyperplasia, and granulation tissue. Comparison of enhancement patterns, T2 signal, ADC values, contour regularity, and absence of diffusion restriction helped differentiate these possibilities. The absence of a discrete mass, the smooth contour, homogeneous enhancement, elevated ADC, and stability on interval follow-up imaging favored a benign non-neoplastic process (Table 1). Biopsy was considered but deferred because no focal lesion was present and the risk of postoperative bleeding was significant.

**Table 1:** Differential diagnosis of oropharyngeal nodular thickening after total laryngectomy and adjuvant radiotherapy, with comparative imaging characteristics.

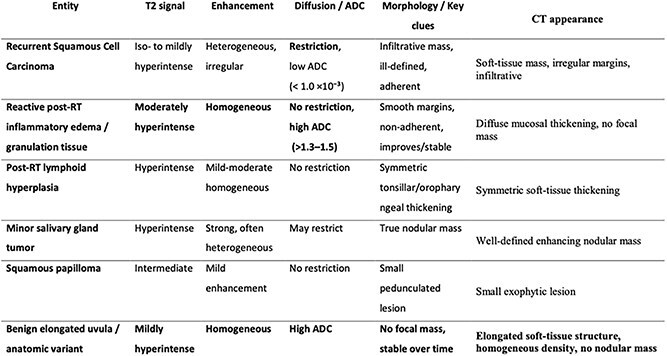

Imaging features from MRI and CT were compared to differentiate recurrent squamous cell carcinoma from benign postoperative and post-radiotherapy conditions. MRI assessment included signal characteristics, enhancement pattern, diffusion-weighted imaging, and approximate ADC values, while CT appearance provided complementary structural information. ADC values represent approximate ranges derived from the literature and should be interpreted in conjunction with imaging morphology and clinical context [3, 4].

**Figure 4:**
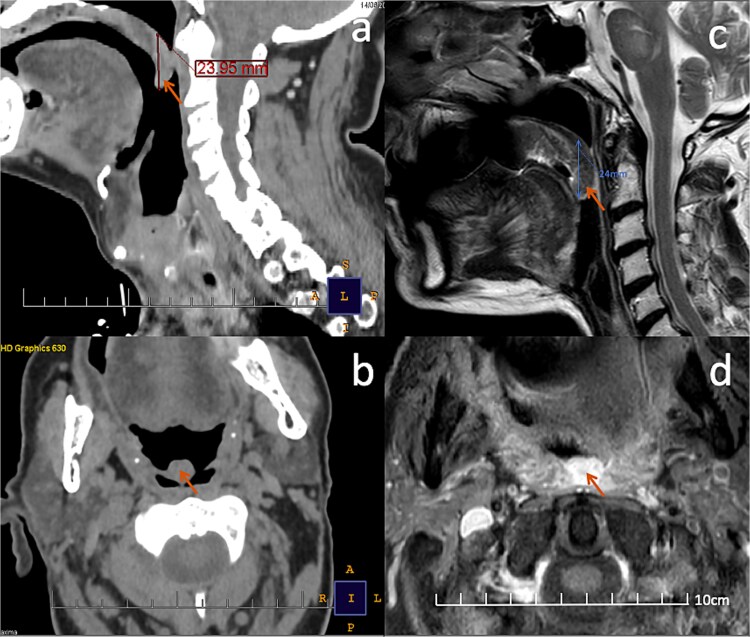
Comparison of pre-operative CT and post-operative MRI. (a, b) sagittal and axial contrast-enhanced CT images demonstrating an elongated uvula (orange arrow) measuring 23.95 mm vs 24 mm in length. (c, d) post-operative sagittal T2-weighted and axial post-contrast T1-weighted MRI acquired through the oropharynx. The uvula (orange arrow) shows homogeneous enhancement without focal mass effect, supporting its benign nature. The length of the uvula remains comparable on both modalities (a vs c); however, a slight increase in transverse diameter is appreciable on axial images (b vs d), likely related to reactive post-surgical inflammatory changes rather than recurrent tumor.

Follow-up sagittal CT and MRI demonstrated stable morphology (Fig. 4), confirming a benign anatomical variant with postoperative inflammatory enhancement rather than malignant recurrence. Adjuvant chemoradiotherapy proceeded without delay, and no recurrence has been observed on subsequent surveillance imaging.

## Discussion

Anatomical variants can present significant diagnostic challenges in post-operative imaging, especially in oncological cases where surveillance for tumor recurrence is paramount. The uvula is a small fleshy structure that plays a crucial role in functions such as swallowing, phonation, and the gag reflex. However, elongated or inflamed uvulas can appear as abnormal masses on imaging, particularly when there is enhancement following gadolinium contrast administration [5].

Several benign conditions, including inflammation (uvulitis), edema, and trauma, can lead to abnormal appearances of the uvula. Such conditions can mimic the appearance of malignancy, as was observed in this case. Studies have documented that elongated uvulas, which are typically longer than 1 cm, can occasionally cause symptoms such as snoring or sleep apnea, but rarely represent a pathological process [5–7].

The use of contrast-enhanced MRI in head and neck cancer surveillance is invaluable, but it is not without limitations. In this case, the enhancement of the uvula raised concerns of a recurrence or a synchronous malignancy, which were later ruled out based on imaging features and clinical correlation. Uvula enhancement is commonly seen in benign processes like inflammation [3–6].

A structured differential diagnosis was considered to avoid premature interpretation as tumor recurrence. Potential differential entities included recurrent squamous cell carcinoma, minor salivary gland tumor, squamous papilloma, post-radiotherapy lymphoid hyperplasia, and granulation tissue. Comparative evaluation of imaging characteristics (T2 signal, contrast enhancement pattern, diffusion restriction and ADC values, and morphologic features) helped distinguish these possibilities (Table 1) [8, 9]. The absence of a focal soft-tissue mass, the smooth and well-defined contour, homogeneous enhancement, elevated ADC values, and stability on interval imaging strongly favored a benign non-neoplastic process rather than any of the aforementioned entities [8, 10].

A thorough understanding of head and neck anatomy and the potential for benign post-surgical changes is essential for accurate interpretation of post-treatment imaging. Clinical correlation, including careful review of patient history and physical examination, remains critical to avoid over-diagnosis or false-positive interpretation. In this case, recognizing the elongated uvula as a benign finding prevented unnecessary diagnostic procedures or additional radiation exposure, which could have had significant implications for the patient’s treatment course and quality of life [11, 12].

To the best of our knowledge, this is the first reported case of an elongated uvula mimicking early recurrence on planning CT in a post-laryngectomy patient undergoing adjuvant radiotherapy. Although elongated uvula is a known anatomical variant, the combination of post-surgical changes, homogeneous tip enhancement on MRI suggesting inflammatory edema, and its presentation as a false-positive lesion during radiotherapy planning is novel. This imaging pitfall has important clinical implications, as it may lead to unnecessary biopsy or treatment interruption.

In practical terms, misinterpretation of this benign finding could result in delays of several weeks in the initiation of adjuvant radiotherapy, additional imaging examinations, or inappropriate treatment escalation.

Awareness of this scenario is therefore essential to prevent misinterpretation and avoid detrimental modification of oncologic management.

To assess the novelty of this case, a structured literature search was performed using PubMed, using combinations of the following keywords: ‘elongated uvula,’ ‘uvula inflammation,’ ‘post-laryngectomy imaging,’ ‘radiotherapy planning CT’, and ‘head and neck cancer imaging pitfalls’. Only a limited number of reports describing uvular abnormalities on post-treatment imaging were identified, and none specifically reported an elongated inflammatory uvula mimicking early tumor recurrence on radiotherapy planning CT. This highlights the originality of the present case. Moreover, this case underscores the importance of careful retrospective image review, particularly in the context of anatomical variants. Prior imaging, including pre-surgical scans and MRI, should be systematically revisited to determine whether a suspected lesion represents a new pathological finding or a pre-existing benign structure. In our patient, the elongated uvula was present prior to surgery and subsequently became inflamed, thereby mimicking a suspicious lesion on post-treatment imaging.

## Conclusion

This case illustrates how benign postoperative changes, such as an elongated uvula, may closely mimic tumor recurrence during radiotherapy planning. In post-laryngectomy patients, systematic comparison with pre-treatment imaging, multiplanar MRI reconstructions, and awareness of benign anatomic variants are essential to avoid false-positive interpretations and prevent unnecessary biopsy or treatment modification. Accurate radiologic assessment supported by clinical correlation remains critical for safe and effective post-treatment management. The patient continues chemoradiotherapy with no evidence of recurrence on imaging follow-up.

### Ethic approval

This case report is exempt from ethical approval according to the guidelines of the Institutional Review Board (IRB) of Ibn Sina University Hospital. According to our institution’s policies, case reports and studies that do not involve clinical trials or clinical interventions are not subject to formal ethical review. Written informed consent was obtained from the patient, including explicit permission for anonymized publication of clinical data and imaging.

## Consent

Written informed consent was obtained from the patient on 19 September 2024, in accordance with ethical standards, for the publication of this case report and images.

## Guarantor

Dr Rania Bouanane.
